# The Art of Packaging the Sperm Genome: Molecular and Structural Basis of the Histone-To-Protamine Exchange

**DOI:** 10.3389/fendo.2022.895502

**Published:** 2022-06-22

**Authors:** Lindsay Moritz, Saher Sue Hammoud

**Affiliations:** ^1^ Department of Human Genetics, University of Michigan, Ann Arbor, MI, United States; ^2^ Department of Obstetrics and Gynecology, University of Michigan, Ann Arbor, MI, United States; ^3^ Department of Urology, University of Michigan, Ann Arbor, MI, United States

**Keywords:** sperm chromatin, histone, epigenetics, chromatin remodeling, histone displacement

## Abstract

Male fertility throughout life hinges on the successful production of motile sperm, a developmental process that involves three coordinated transitions: mitosis, meiosis, and spermiogenesis. Germ cells undergo both mitosis and meiosis to generate haploid round spermatids, in which histones bound to the male genome are replaced with small nuclear proteins known as protamines. During this transformation, the chromatin undergoes extensive remodeling to become highly compacted in the sperm head. Despite its central role in spermiogenesis and fertility, we lack a comprehensive understanding of the molecular mechanisms underlying the remodeling process, including which remodelers/chaperones are involved, and whether intermediate chromatin proteins function as discrete steps, or unite simultaneously to drive successful exchange. Furthermore, it remains largely unknown whether more nuanced interactions instructed by protamine post-translational modifications affect chromatin dynamics or gene expression in the early embryo. Here, we bring together past and more recent work to explore these topics and suggest future studies that will elevate our understanding of the molecular basis of the histone-to-protamine exchange and the underlying etiology of idiopathic male infertility.

## Introduction

Spermatogenesis ensures transmission of genetic information to the next generation by maintaining male fertility throughout life. Three biologically distinct processes safeguard the continuous generation of sperm: mitosis, meiosis, and spermiogenesis – the last of which involves extensive remodeling of both cytoskeleton and chromatin to achieve a significant compaction of the sperm head (1, 2). At both a molecular and structural level, sperm chromatin is highly distinct from the chromatin in oocytes and somatic cells. While nucleosome-based packaging by histone octamers produces a bead-on-a-string structure of chromatin in oocytes and somatic cell nuclei, the sperm genome is packaged by small, arginine-rich basic proteins known as protamines (P1 and P2), which presumably package the DNA into toroidal structures leading to a 10-fold greater chromatin compaction state than the somatic cell nucleus (2–4). This differential packaging program evolved over 500 million years ago, yet its biological and evolutionary significance remains unknown.

Seminal work used biochemical and genetic approaches to identify intermediate proteins involved in the histone-to-protamine transition; however despite its biological importance, our insight into how chromatin-associated factors/remodelers are involved remains limited (5–9). We lack both genetic and molecular reagents to identify chromatin-associated factors as well as *in vitro* experimental systems to investigate mechanisms. In this review, we summarize the current understanding of chromatin dynamics during spermiogenesis and the advances made to understand sperm chromatin 3D organization. A greater understanding of sperm genome packaging and molecular organization will inform our understanding of how this process is dysregulated in infertility and will aid in the development of clinical assays and therapeutic approaches that may enhance clinical care and reproductive outcomes.

## Chromatin Dynamics During Spermatogenesis Lead to a Unique Packaging Mechanism of Sperm Chromatin

The histone-to-protamine transition is one of the most poorly understood aspects of spermiogenesis and the sequence of events is also known to vary across species. However, this remodeling process is believed to occur in a stepwise fashion, wherein canonical histones are sequentially replaced by testis-specific histone variants (10–13) followed by transition proteins (TNPs) (14–16) and finally by protamines (17). These sequential events are thought to loosen histone-DNA interactions, thereby facilitating histone removal and permitting protamine incorporation.

### Mechanisms Contributing to Nucleosome Destabilization: Histone Variants and Histone Post-Translational Modifications

**Figure 1:**
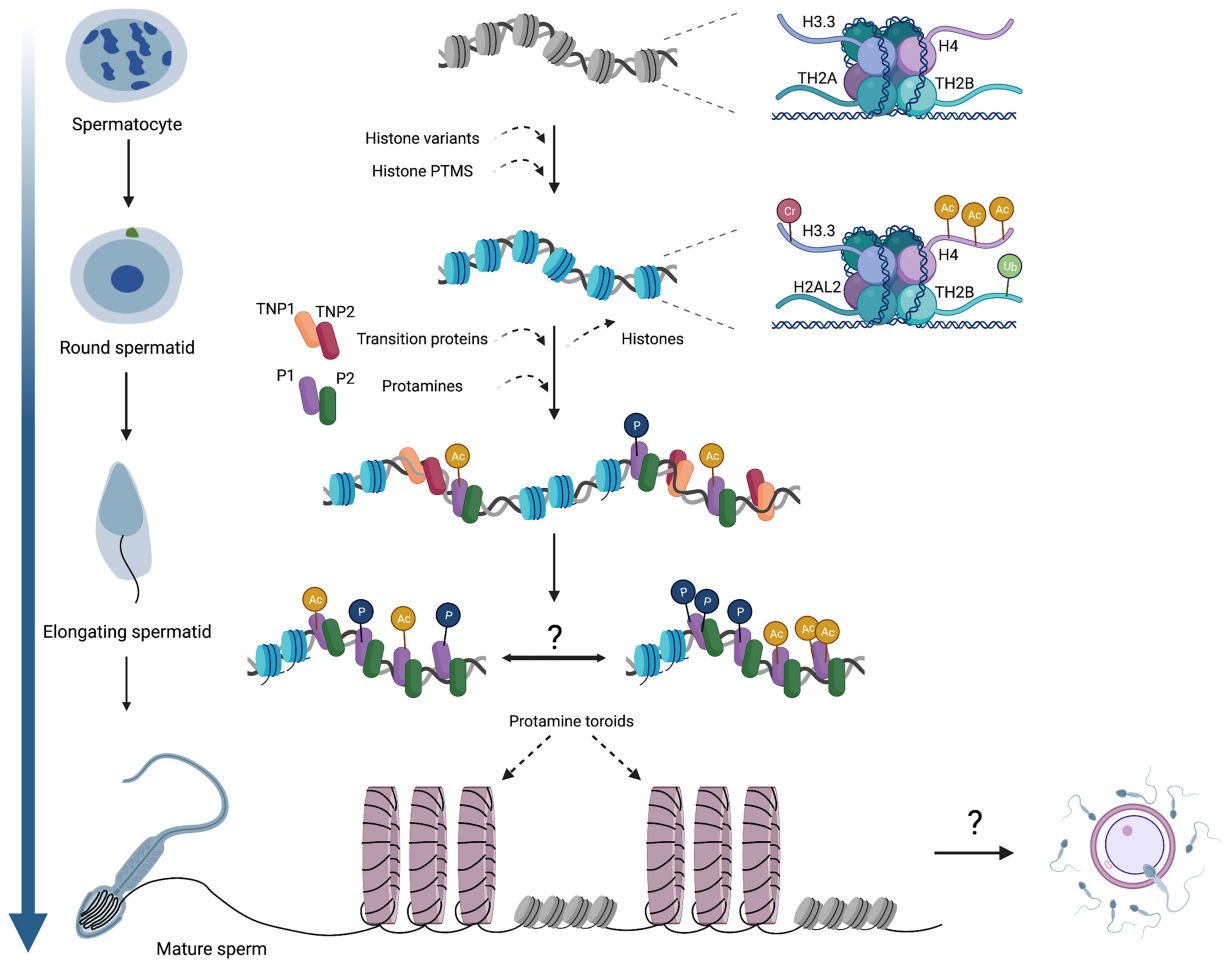
An overview of chromatin dynamics and intermediate stages of the histone-to-protamine exchange. Many histone variants are incorporated in meiotic spermatocytes, including H3.3, TH2A, and TH2B. Histone variant incorporation continues in post-meiotic round spermatids (H2AL2), concomitant with various histone PTMs that induce nucleosome destabilization. As spermatids begin elongation, TNPs and protamines are expressed and incorporated onto chromatin, but whether these act as discrete steps or co-occur remains unknown. It is also established that protamines acquire various PTMs, but the genomic localization of these PTMs (i.e. whether they occur randomly throughout the genome or localize into discrete domains) has not been determined. Ultimately, protamine-DNA binding forms toroidal structures of sperm chromatin, making sperm chromatin distinct from that of both oocytes and somatic cells. The contribution of sperm chromatin structure and the sperm epigenome to embryonic development will also be a fascinating area for future exploration. Cr,crotonylation; Ac, acetylation; Ub, ubiquitination; P, phosphorylation.

The hallmark of spermiogenesis is the dramatic reorganization of chromatin in spermatids, in which most histones are sequentially replaced with protamines (**Figure 1**) (5, 6, 18). To achieve this reorganization, the spermatid nucleus undergoes a series of intermediate state transitions, including the incorporation of histone variants (H1t, H2A.X., H2A.Z., H3.3, H3t, TH2A, TH2B) – many of which are testis-specific – during meiosis (19–26) and throughout post-meiotic maturation in round spermatids (H2AL.1/2, HILS) (11, 12, 27, 28). Several *in vitro* studies have demonstrated that the incorporation of histone variants such as H3T, H2AL2, and TH2B induces nucleosome destabilization by altering histone-DNA binding and weakening the associations between H2A/H2B dimers and H3/H4 tetramers, to ultimately promote reorganization of the chromatin (10, 29–31).

Although histone variants are presumed to instruct the chromatin remodeling process, inferring specific roles of these complexes in the exchange process through the analysis of gene loss of function phenotypes is sometimes challenging due to confounding functions outside of the histone-to-protamine exchange. For instance, knockout of H3T results in defective spermatogonial differentiation, ultimately leading to azoospermia (26). On the other hand, H2AL2 knockout males are infertile and exhibit defective genome packaging during spermiogenesis (12). High-resolution electron microscopy (EM) analysis of chromatin compaction in H2AL2 knockout sperm identified more diffuse packaging and many translucent areas, indicative of defective global genome compaction. This defect is due to inefficient assembly of both TNPs and protamines onto chromatin, raising the question of how a histone variant, functioning upstream of both transition proteins and protamines, prevents their proper incorporation onto chromatin (12). However, not all variants incorporated during spermiogenesis are essential for the histone-to-protamine exchange. For example, mice lacking TH2B are fertile because TH2B loss is compensated for by the retention of alternative H2B isoforms and the addition of destabilizing PTMs such as arginine methylation and lysine crotonylation within the histone fold domains of H2A, H2B, H3, and H4, as opposed to the histone tail (25). Similarly, mice lacking the testis-specific linker histone H1t retain alternative H1 isoforms and are fertile (32–34). Therefore, the differences in cellular phenotypes reported for each of the histone variants may be attributed to gene family expansions and the extent to which protein variants have retained ancestral or acquired novel functions. A greater understanding of histone variant evolution and phylogeny may help us predict and/or reconcile reported phenotypes for the different proteins involved in germ cell development and packaging (35–37).

In addition to nucleosome destabilization by the incorporation of histone variants, histone PTMs can alter histone-DNA binding dynamics and aid in promoting chromatin accessibility (**Figure 1**). Preceding the histone-to-protamine exchange, well-documented histone hyperacetylation mechanisms promote chromatin accessibility by inhibiting folding of nucleosomes into chromatin fibers (38–44). Accordingly, genetic knockout of either EPC1 or Tip60, two components of the mammalian NuA4/TIP60 nucleosome acetyltransferase complex, results in a global decrease in H4 hyperacetylation, leading to aberrant spermatid elongation, decreased TNP2 incorporation, and ultimately impaired fertility (45). Similarly, the loss of GCN5, another histone acetyltransferase, in differentiating spermatogonia (using *Stra8-Cre*) leads to aberrant spermatid development and impaired fertility (46). Indicative of defects in remodeling and compaction, conditional GCN5 mutant sperm feature morphological abnormalities such as rounded or blunted triangular-shaped heads. Chromatin characterization further reveals an increase in histone retention and concomitant decrease in sperm protamine levels (46). In related work, loss of the chromatin reader BRDT–which directly binds to acetylated histones and facilitates their removal, thereby initiating repackaging of the genome during spermiogenesis– results in decreased chromatin compaction in spermatids, aberrant spermatid elongation, decreased sperm counts, and infertility (47, 48). Together, these studies illustrate that targeted disruption of histone acetylation writers and readers leads to similar phenotypes, underscoring the importance of histone acetylation for histone-to-protamine exchange and sperm function.

Although H4 hyperacetylation is a well-established modification known to precede the histone-to-protamine exchange in multiple species, other modifications, such as di-and trimethylated H3K79, catalyzed by DOT1L, have been reported to temporally overlap with H4 hyperacetylation in both human and mouse spermatids (49, 50). H3K79me3 is enriched at the chromocenter (the constitutive heterochromatin) of round spermatids and at repetitive elements in mESCs, whereas H3K79me2 accumulates at euchromatic regions, often downstream of promoters of actively transcribed genes (51–55). DOT1L loss of function mutants are embryonic lethal (56), therefore preventing the analysis of H3K79 methylation in the histone-to-protamine exchange or spermatid-specific cellular functions. Therefore, a round spermatid-specific conditional knockout of DOT1L or H3K79 point mutant mice will be needed to dissect the impact of the K79 residue or its methylation during spermiogenesis. In related work, histone crotonylation, a newly identified modification, is reportedly enriched in elongating spermatids concomitant with H4 hyperacetylation (49, 57). Histone crotonylation in somatic and germ cells is enriched at TSSs, and largely overlaps with active histone modifications (57). Consistent with a possible role for crotonylation in the histone-to-protamine exchange, CDYL (chromodomain Y-like protein, an eraser of crotonylation) knockout mice exhibit reduced levels of chromatin-bound transition proteins, sperm motility defects, and decreased fertility (58). Given the general enrichment in spermatids for modifications canonically associated with transcriptional activation in somatic cells, together with the well-documented pervasive transcription observed in round spermatids (59–61) and the lack of reported phenotypes for many spermatid-specific expressed genes, this begs the question of whether the physical process of gene transcription may be important in nucleosome destabilization and subsequent exchange–a hypothesis that will need to be evaluated in future studies (62–65).

### Transition Proteins

Transition proteins are present in many species including mouse, rat, human, ram, and boar (20, 66, 67). Two major TNPs–TNP1 and TNP2– are prominent in rodent spermatids (68). TNP1 is highly expressed (~2.5x higher in spermatids than TNP2) and conserved in various mammals, while TNP2 sequences are poorly conserved across species and its expression level and protein abundance vary between species (20, 67, 69, 70). Knockout of TNP1 results in male sub-fertility, and sperm exhibit abnormal morphology and decreased progressive motility (71). A detailed analysis of sperm chromatin from TNP1^-/-^ sperm reveals alterations in protein composition–including a compensatory increase in TNP2 in mature sperm as well as an accumulation of unprocessed P2 (71). Interestingly, fertility in TNP2^-/-^ males is unaffected, although progressive sperm motility is decreased, and sperm morphology is slightly abnormal. Like TNP1^-/-^ males, TNP2^-/-^ males also exhibit an increased level of unprocessed P2 in mature sperm. In both TNP1^-/-^ and TNP2^-/-^ males, defects in progressive sperm motility did not impact fertilization rates, as assessed by blastocyst formation resulting from intracytoplasmic sperm injections (ICSI) (72). However, double knockout mice are completely infertile, with a near complete loss of progressive sperm motility and alterations in sperm chromatin composition (72), underscoring the importance of these proteins in finetuning chromatin packaging.

Previous dogma posited that TNPs are incorporated onto chromatin following histone eviction and occupy the majority of the genome in elongating spermatids, thereby acting as intermediates between histones and protamines (73). This initial assumption was based on the knowledge that the two transition proteins- TNP1 and TNP2, are both relatively small and highly basic, with high lysine (~19%) and arginine (~21%) content, that can mediate electrostatic interactions with the phosphate backbone of DNA uniformly along the TNP molecules (74). However, accumulating molecular, genetic, and biochemical data suggest that TNPs may not replace histones completely as initially predicted by the stepwise model.

First, numerous studies observed that transition protein expression does not precede that of protamines, but rather they are co-expressed in spermatids along with other histone variants and can be directly visualized in the spermatid nucleus in specific spermatogenic stages (IX-I, **Figure 1**) (12, 69, 75). This observation suggests the possibility that these proteins act in concert, rather than sequentially, to ensure successful chromatin remodeling. Interestingly, early *in vitro* data shows that TNP1 has a >8-fold affinity for single-stranded DNA (ssDNA) over double-stranded DNA (dsDNA), and in contrast to H1 histone, TNP1 forms less stable structures with DNA even at higher ionic strength (50 mM NaCl), which is still below physiological salt concentrations (76). In contrast, TNP2 has a 40X higher affinity for dsDNA and stabilizes and condenses DNA fibers *in vitro* at a broad range of ionic strengths (77, 78). These observations reveal that DNA binding and stabilizing properties of TNP1 and 2 differ greatly, suggesting that it is unlikely that TNP1 binds dsDNA, but rather it may intercalate between nucleic acid bases resulting in local melting of the DNA duplex, while TNP2 physically replaces histones. However, recent nucleosome invasion assays show that TNP2 does not physically replace canonical nucleosomes or testis-specific variant-containing nucleosomes, but rather TNP2 intercalates the nucleosome, leading to nucleosome destabilization/eviction or TNPs may serve as a scaffold on histones to aid in protamine recruitment/deposition onto chromatin (12). Therefore, various categories of nuclear proteins (histone variants, transition proteins, protamines), act in a concerted manner to mediate a direct transition from histone-to-protamine states, as observed in certain species of birds and fish (79, 80). The differences in the complexity of the remodeling process are intriguing and makes us wonder whether these differences may be due to biochemical and biophysical properties of protamine proteins themselves or whether analogous proteins (variants and TNPs) with similar properties are needed in other species but have not yet been identified.

### Protamines

During spermiogenesis, small, sperm-specific, and highly arginine-rich protamines serve to compact paternal DNA, allowing the sperm head to adopt a highly condensed, hydrodynamic shape that protects the paternal DNA during transit to the egg (81, 82). Most mammals, including mice and humans, express two forms of protamine: protamine 1 (P1) and protamine 2 (P2). Rapidly evolving across species (6, 83–86), protamines are subject to strong positive selection that tightly maintains arginine/serine-rich regions, but not strict sequences (85–87). Whether protamines are possibly coevolving with the DNA sequence or if protamines from different species have different binding affinities to certain genomic regions within and across species remains to be determined.

P1 is expressed in its mature form, while P2 is initially expressed as a longer precursor (pro-P2) and undergoes selective proteolytic processing to produce its mature form (P2) once bound to DNA (88, 89). Truncation of the amino terminus of P2, the portion of the protein that is typically cleaved (cP2) in the nucleus, causes infertility due to inefficient import of the protein into the nucleus, resulting in altered protamine ratios and immotile sperm; suggesting that the longer isoform may be required for protamine-chaperone interactions (90).

Across species, the P1:P2 ratio is highly variable but maintenance of a species-specific P1:P2 ratio is critical for normal fertility (91–94). Conversely, alterations in protamine ratios in mice and humans are associated with increased sperm DNA fragmentation, diminished fertilization rates, and defects in sperm morphology and motility (12, 90, 92, 95). Consistent with the importance of P1:P2 ratio correlations, initial haploinsufficiency studies of either P1 or P2 genes resulted in infertility (96). However, subsequent studies using CRISPR-Cas9 engineered P1 or P2-deficient mouse lines found that haploinsufficiency of P1 is sufficient to cause infertility, whereas loss of one P2 allele is tolerated and complete deletion is necessary to cause infertility (97, 98). Together, these results suggest that a defined composition of chromatin is necessary for fertility, and deviations have negative consequences.

Given that protamines were assumed to bind uniformly in the genome and not believed to bear PTMs, their potential role as informational carriers has been largely overlooked. Recent biochemical and mass-spectrometry analysis by us and others led to the discovery that P1 and P2 proteins from mature sperm carry multiple PTMs, including phosphorylation, acetylation, and methylation (95, 99). Dynamic phosphorylation/dephosphorylation of protamines was previously suggested to have a role in modulating protamine-DNA dynamics in a variety of species (100–103). Analysis of radiolabeled proteins from mouse and rat seminiferous tubules by acid urea gel electrophoresis revealed that newly synthesized protamines are phosphorylated and subsequently dephosphorylated shortly after their deposition onto DNA (88), a phenomenon also observed in human sperm (101, 102). More recent studies reported comprehensive catalogs of mouse and human protamine PTMs, with ~53% of P1 peptides in mouse containing PTMs and ~16% of P2 peptides (99, 104). Importantly, the sites of protamine modifications are maintained within a species but not conserved across species, suggesting that these modifications may confer a lineage-specific function (95). The identification of protamine PTMs was surprising since these proteins are placed onto DNA after meiosis and during spermatid maturation–when all transcription in germ cells has halted, suggesting that these modifications have no effect on spermatid gene expression. Rather, these modifications may be required for either 1) mediating protamine protein deposition onto DNA and/or regulating sperm genome packaging, 2) conveying epigenetic information to the zygote, or 3) instructing paternal genome chromatin reorganization.

Indeed, recent studies suggested that protamine phosphorylation during spermiogenesis is important for modulating protamine-DNA dynamics and maximizing chromatin compaction (105, 106). Recently, Gou et al. reported that phosphorylation of serine residues in P1 during early embryogenesis is required to weaken protamine-DNA interactions, thereby permitting male pronuclear remodeling and protamine-to-histone exchange (106). Additionally, we found that loss of acetylation at P1 lysine (K) 49 drastically alters sperm chromatin composition and results in subfertility in the mouse, premature dismissal of P1 from paternal chromatin in the zygote and altered DNA compaction and decompaction rates *in vitro* (95). Together, these studies establish a regulatory role for protamine PTMs in governing sperm chromatin packaging and unpacking in the embryo. Whether PTMs on human protamines similarly influence these processes remains to be determined. Additionally, assessing whether alterations in protamine PTM levels affect embryonic gene expression, as is the case for alterations in histone levels/PTMs, will further provide insight into the function of these modifications *in vivo.*

Although the histone and protamine packaging systems were discovered decades ago, we know relatively little about whether protamine protein placement varies along the sperm genome and how they are placed onto DNA, relative to the wealth of data available for histone proteins. The current models suggest that protamine proteins bind uniformly throughout the genome, but definitive data to support or refute such a model are lacking. The super-condensed protamine-packaged chromatin state does not easily lend itself to mechanistic investigations. Moreover, the scarcity of lysine residues in protamines makes it difficult to crosslink protamine proteins and DNA to prevent protamine on/off dynamics, which can lead to non-biological associations during sample processing.

## Chromatin Remodelers Involved in Histone-To-Protamine Exchange

Studies of chromatin-associated factors/remodelers involved in sperm chromatin remodeling are hampered by the lack of genetic and molecular reagents with which to identify chromatin-associated factors *in vivo* and the lack of experimental systems to model the histone-to-protamine exchange process *in vitro*. However, candidate gene knockout studies have begun to shed insights. For example, in a full body knockout of Chromodomain Helicase DNA Binding Protein 5 (CHD5), with phenotypes ranging from subfertility to infertility, the infertility is not caused by changes in the hypothalamic pituitary axis or somatic cell numbers. Instead, the infertility appears to be germ cell-intrinsic; presenting as defects in spermatid elongation and condensation defects, consistent with CHD5 expression in steps 7-10 of spermatid maturation, immediately preceding and overlapping with the extensive chromatin remodeling (107, 108). Biochemical fractionation of spermatids shows that CHD5 deficiency perturbs histone hyperacetylation and the histone-to-protamine transition, leading to aberrant retention of histones and elevated levels of transition proteins and protamines (107, 108). The overall higher level of protamine mRNA and protein expression in CHD5^-/-^ males, assessed by qPCR and immunoblotting, indicates a possible role for CHD5 in protamine transcriptional and/or translational control (107).

Other studies have explored the roles of ATP-dependent chromatin remodeling complexes SWI/SNF (SWItch/Sucrose Non-Fermentable) and ISWI (Imitation SWItch). A knockout of BRG1 (a SWI/SNF component and transcription activator) in germ cell progenitors resulted in a mid-pachytene arrest, preventing investigations in post-meiotic round spermatids (109). The zinc finger and bromo-domain protein ACF1/BAZ1A, a component of ISWI, binds to the chromatin remodeler SNF2H and plays an essential role during post-meiotic spermiogenesis, as evidenced by its deletion resulting in infertility with increased DNA damage and spermiation defects (110). At a general level, deletion studies are confounded by upstream functions in spermatogenesis, making it difficult to investigate the specific role of chaperones/remodelers in nucleosome eviction/protamine deposition and to discern whether histone removal and protamine deposition are functionally distinct processes that require unique or shared proteins. As the process of spermiogenesis occurs within the testis, and its byproduct is sperm DNA compaction, monitoring the remodeling process in a living organ is not possible. However, the combination of future targeted proteomic analyses with an *in vitro* chromatin remodeling system holds promise for identifying candidate remodelers and uncovering molecular details of their roles in the histone-to-protamine exchange.

## Something Old, Something New: Experimental Approaches to Understand Sperm Structure and 3D Organization

Decades of *in vitro* biochemistry and biophysics experiments have provided fundamental insights into protamine-DNA interactions and the structure of sperm chromatin imposed by protamine binding. Early *in vitro* studies primarily relied on measuring the behavior and properties of either polyarginine/polylysine peptides or purified salmon or bull (domestic cattle, *Bos taurus*) sperm protamine (111–116). Raman and nuclear magnetic resonance (NMR) spectroscopy using a polyarginine (R6WGR6) peptide – a representative sequence of the central arginine-rich domain of bull P1 – suggested that protamines bind preferentially to the major groove of DNA, with one protamine molecule bound per turn of the helix (117). Using Particle Induced X-ray emission, *in vivo* measurements of the total amount of nuclear phosphorous and sulfur in sperm from various species estimated that bull P1 binds ~10-11 base pairs of DNA. Assuming that the P1 binding to DNA mode is conserved across species, and given known P1:P2 ratios, calculations of phosphorous:sulfur ratios predict that P2 binds ~15 base pairs, although the exact footprints of P1 and P2 remain to be determined (115).

Several early studies examining the 3-dimensional topology of the sperm genome indicated that sperm DNA, like somatic cell DNA, forms loops, as inferred by the formation of nuclear “halos” when sperm are treated with SDS and stained with ethidium bromide (118–121). The loops formed by hamster sperm were noted to be smaller than those found in somatic cell nuclei by ~60%, and to consist of ~50 kb of DNA on average. Furthermore, these loops are anchored to a structural component of the sperm nucleus – termed the nuclear matrix – at attachment sites known as matrix attachment regions, or MARs (120–126). When isolating DNA loops or nuclear matrices and analyzing the localization of a handful of genes, early data suggested that the 5SRNA gene enriches at the nuclear matrix, while satellite DNA is detected in loops (120, 125, 127, 128), suggesting that DNA organization and sites of DNA attachment to the matrix may not be random, but programmatic. However, future studies are needed to explore such assumptions genome-wide and determine whether MARs are associated with specific DNA sequences or with specific chromatin (histone or protamine-bound) in sperm.

The molecular nature of sperm genome organization was initially difficult to resolve because sperm decondensation by chemical agents was necessary to visualize sperm DNA, which prevented the investigation of the structure of unperturbed sperm chromatin *in vivo.* However, by examining intact native sperm or reconstituted salmon sperm protamine with either lambda phage DNA or linearized plasmid DNA, using a variety of techniques including light scattering (129, 130), electron and atomic force microscopy (116, 131), fluorescence microscopy (132, 133), and DNA elasticity measurements (134), it was discovered that protamine-DNA complexes *both in vivo* and *in vitro* were organized into toroidal structures. The identification of a toroid is intriguing given that other positively charged molecules, including hexamine-cobalt (III), spermine, and spermidine, have also been shown to form DNA toroids (135, 136). While toroids are the identified packaging unit, the exact mechanism of folding and unfolding of the toroid is unknown, but presumed to be mediated by single loops coming together and then separating back out. Recent studies using tethered particle motion assays and AFM found that salmon protamine uses a multi-step process, forming multiple independent loops of a roughly defined diameter that come together before forming a larger toroidal structure (137). Furthermore, the formation of these structures relies on protamine binding-and-bending the DNA, whereby multiple protamine molecules bind locally to a DNA segment to induce bending of the DNA filament to form loops (138). These data are in agreement with previous studies that identified loops formed by sperm DNA *in vivo* (119, 121, 124) as well as our recent EMSA and single molecule DNA curtain assays, which suggest that large-scale genome compaction by protamines is achieved by protamine protein cooperativity (95). Although these experiments provide a basic foundation of knowledge of sperm genome packaging, these data rely on protamines from teleost fish or bull P1 proteins, which are highly divergent from both mouse and human protamines in both sequence and amino acid composition. Therefore, we are currently presuming that protamines from all species display a stereotypic random association with DNA that is sequence-independent. Future studies utilizing mammalian proteins or multiple protamine protein proteoforms (P1, 2, and/or 3) are needed to explore whether packaging is universal regardless of source or combination of proteins used. By learning how protamines guide sophisticated genome self-assembly, one may utilize the inherent rules of cellular machineries to synthesize designer molecular structures *in vitro* which can be used for gene therapy delivery.

Multiple groups have taken advantage of chromatin capture assays to allow high-resolution mapping of the 3D organization of not only sperm chromatin across a variety of species, but also of pre-implantation embryos, providing foundational insight into sequence-level 3D chromatin organization from gametes to the next generation. Initial Hi-C studies in mouse sperm curiously found that despite sperm being packaged by protamines, sperm 3D organization resembles both fibroblast (139) and mESC (140) genome organization, with the exception that sperm from mouse and macaque possess a significant number of long-range interactions (>2 Mb), with a significant fraction of these interactions being between TADs (141, 142). Likely, these extra-long-range interactions aid in either establishing or maintaining the hypercondensed state of the sperm nucleus. In contrast, zebrafish sperm, which completely lack protamines, lack TADs altogether, and resemble mitotic chromosomes. Contact matrices exhibit “flare-like” structures, indicative of clustering of large extended genomic loops at a set point that is equidistant for all loops (143). Analysis of these flares illustrated that zebrafish sperm do indeed display periodic domains of ~150 kb that repeat every 1-2 mega bases–a chromatin structure resembling the mitotic cell chromatin landscape, and suggesting that the overall 3D chromatin architecture of the zebrafish sperm genome may be distinct from protamine-bound sperm genomes (144). However, since the 3D chromatin structure of a zebrafish sperm, which is fully packaged in histone, is different from somatic cells, this begs the question of whether the published structures of mammalian sperm, which resemble somatic cells and mESCs, are truly representative of the *in vivo* architecture. Given its hyper-condensed state, the protamine-packaged genome is poorly accessible to restriction enzymes. Therefore, applying current Hi-C technology in mammalian sperm is likely to be particularly technically challenging, requiring methodological innovations before Hi-C can be leveraged towards generating a comprehensive view of *the in vivo* sperm genome architecture.

## Conclusions and Future Perspectives

Protamine-based compaction of paternal DNA and the unique sperm chromatin state have fascinated scientists for decades. We have gained foundational knowledge about the histone-to-protamine transition, yet, we still lack a comprehensive understanding of the mechanisms governing critical steps of the exchange process. Specifically, it remains unknown which specific factors are required for histone eviction/protamine deposition and importantly, how all basic proteins function together to ensure successful exchange. Future studies examining whether histone variants, transition proteins, and protamines truly function as independent intermediates or act in combined mechanisms will shed light on the regulation of this process and inform development of targeted interventions to treat infertility. The recent discovery of protamine PTMs suggest that nuanced interactions may control aspects of the exchange process and chromatin condensation during spermiogenesis, but whether these modifications constitute a species-specific code analogous to the histone code for instruction of development remains to be determined. Lastly, while both classical and modern approaches have been applied towards understanding the structure of sperm chromatin, structure determination by cryo-EM will undoubtedly provide a more complete picture. These future studies will not only significantly increase our understanding of sperm genome packaging, but may aid in our understanding of idiopathic male infertility or eventually lead to the development of clinical assays that can better predict reproductive success.

## Author Contributions

LM contributed to manuscript writing and revision. SH contributed to manuscript writing and revision. All authors contributed to the article and approved the submitted version.

## Funding

Research in the Hammoud lab is supported by National Institute of Health (NIH) grants 1R21HD090371-01A1 (SSH), 1DP2HD091949-01 (SSH), R01HD104680 (SSH), training grants NSF 1256260 DGE (LM), Rackham Predoctoral Fellowship (LM), T32GM007315 (LM), and Open Philanthropy Grant 2019-199327 (5384) (SH).

## Conflict of Interest

The authors declare that the research was conducted in the absence of any commercial or financial relationships that could be construed as a potential conflict of interest.

## Publisher’s Note

All claims expressed in this article are solely those of the authors and do not necessarily represent those of their affiliated organizations, or those of the publisher, the editors and the reviewers. Any product that may be evaluated in this article, or claim that may be made by its manufacturer, is not guaranteed or endorsed by the publisher.
